# Does calcium diffusional global feedback leads to slow light adaptation in *Drosophila* photoreceptors? - A 3D biophysical modelling approach

**DOI:** 10.1186/1471-2202-12-S1-P56

**Published:** 2011-07-18

**Authors:** Zhuoyi Song, Marten Postma, Weiliang Chen, Daniel Coca, SA Billings, Roger C Hardie, Mikko Juusola, Erik De Schutter

**Affiliations:** 1 Department of Biomedical Science, University of Sheffield, Sheffield S10 2TN, UK; 2 Department of Automatic Control and Systems Engineering, University of Sheffield, Sheffield S1 2TN, UK; 3 Department of Physiology, Development and Neuroscience, University of Cambridge, Cambridge CB2 3DY, UK; 4 State Key Laboratory of Cognitive Neuroscience and Learning, Beijing Normal University, Beijing 100875, China; 5 Computational Neuroscience Unit, Okinawa Institute of Science and Technology, Okinawa 904-0411, Japan; 6 Theoretical Neurobiology, University of Antwerp, 2610 Antwerp, Belgium

## 

*Drosophila* photoreceptors transduce light signals to voltage responses. In natural environment, light intensity can change over 10 log range, whereas the coding range of photoreceptors is only about 50 mV. Thus, to reliably represent natural light changes photoreceptors rely upon powerful adaptation. How do different molecular mechanisms of phototransduction enable light adaptation?

Insect photoreceptors are highly polarized, containing light-sensitive and light-insensitive parts of separate functions. The light-sensitive rhabdomere contains a matrix of 30,000 independent phototransduction units (microvilli), which capture and covert light energy into bursts of currents, fluxing through TRP/TRPL channels inside the microvilli. The light insensitive cell-body, which contains voltage-gated channels, then shapes up the resulting voltage responses.

Recently, we produced a two-compartmental biophysical model of a *Drosophila* photoreceptor [1]. Using this model we showed that the dynamic ratio of the used and unused microvilli (ultrastucture), their stochastic reactions and calcium feedbacks cause fast adaptation of the responses, while a global feedback though the membrane voltage compresses the responses to their limited size. However, this model fails to capture slow adaptive changes in the data, which could be important for adjusting the photoreceptor dynamics to changing image statistics in a more Bayesian way.

As a first step, we wish to test whether calcium could diffuse from the responding microvillus to its neighbours, affecting the speed and sensitivity of their separate reaction cascades. We call this hypothetical cross-talk between microvilli global-diffusional-calcium feedback, and ask if it could induce the slow adaptation.

To investigate this question, we extend our biophysical model of *Drosophila* photoreceptor to a 3D-reaction-diffusion model; taking into account the 3D diffusion of calcium. Here, the signalling molecules are loaded in a reconstructed macro-cell structure, described by a 3D tetrahedral mesh. The reaction pathways are simulated using STEPS (STochastic Engine for Pathway Simulation), based on Gillespie’s Stochastic Simulation Algorithm, extended for diffusion.
